# Prediction of Response to Neoadjuvant Chemoradiotherapy by MRI-Based Machine Learning Texture Analysis in Rectal Cancer Patients

**DOI:** 10.1007/s12029-019-00291-0

**Published:** 2019-08-27

**Authors:** Sajad P. Shayesteh, Afsaneh Alikhassi, Farshid Farhan, Reza Gahletaki, Masume Soltanabadi, Peiman Haddad, Ahmad Bitarafan-Rajabi

**Affiliations:** 1 Department of Physiology, Pharmacology and Medical Physics, Faculty of Medicine, Alborz University of Medical Sciences, Karaj, Iran; 2 Department of Radiology, Cancer Institute of Iran, Tehran University of Medical Sciences, Tehran, Iran; 3 Radiation Oncology Research Center, Cancer Institute, Tehran University of Medical Sciences, Tehran, Iran; 4 Radiation Oncology Department, Cancer Institute, Tehran University of Medical Sciences, Tehran, Iran; 5 Department of Nuclear Medicine, Faculty of Medicine, Shahrekord University of Medical Sciences, Shahrekord, Chaharmahal and Bakhtiari Iran; 6 Cardiovascular Intervention Research Center, Rajaie Cardiovascular Medical and Research Center, Iran University of Medical Sciences, Tehran, Iran; 7 Echocardiography Research Center, Rajaie Cardiovascular Medical and Research Center, Iran University of Medical Sciences, Tehran, Iran

**Keywords:** MRI, Rectal cancer, Radiomics, Machine learning

## Abstract

**Introduction:**

Neoadjuvant chemoradiotherapy (nCRT) followed by surgical resection is the standard treatment for locally advanced rectal cancer (LARC). Radiomics can be used as noninvasive biomarker for prediction of response to therapy. The main aim of this study was to evaluate the association of MRI texture features of LARC with nCRT response and the effect of Laplacian of Gaussian (LoG) filter and feature selection algorithm in prediction process improvement.

**Methods:**

All patients underwent MRI with a 3T clinical scanner, 1 week before nCRT. For each patient, intensity, shape, and texture-based features were derived from MRI images with LoG filter using the IBEX software and without preprocessing. We identified responder from a non-responder group using 9 machine learning classifiers. Then, the effect of preprocessing LoG filters with 0.5, 1 and 1.5 value on these classification algorithms’ performance was investigated. Eventually, classification algorithm’s results were compared in different feature selection methods.

**Result:**

Sixty-seven patients with LARC were included in the study. Patients’ nCRT responses included 11 patients with Grade 0, 19 with Grade 1, 26 with Grade 2, and 11 with Grade 3 according to AJCC/CAP pathologic grading. In MR Images which were not preprocessed, the best performance was for Ada boost classifier (AUC = 74.8) with T2W MR Images. In T1W MR Images, the best performance was for aba boost classifier (AUC = 78.1) with a *σ* = 1 preprocessing LoG filter. In T2W MR Images, the best performance was for naive Bayesian network classifier (AUC = 85.1) with a *σ* = 0.5 preprocessing LoG filter. Also, performance of machine learning models with CfsSubsetEval (CF SUB E) feature selection algorithm was better than others.

**Conclusion:**

Machine learning can be used as a response predictor model in LARC patients, but its performance should be improved. A preprocessing LoG filter can improve the machine learning methods performance and at the end, the effect of feature selection algorithm on model’s performance is clear.

## Introduction

Colorectal cancer (CRC) is the third most common cancer worldwide, the second leading cause of cancer deaths in 2016 in the USA and is slightly more common in men. Rectal cancer accounts for one-third of all colorectal cancers and approximately 39,220 new cases of rectal cancer with an estimated 5-year overall survival rate of 65% occur each year [1–5]. Nowadays, nCRT followed by surgical resection is the standard treatment, which is widely used for treatment of locally advanced (cT3, 4 and/or N+) rectal cancer (LARC) [6, 7]. Prediction of response to treatment has a significant role in selection of treatment approach. Response to nCRT is an important prognostic factor but significantly varies among patients [8].

Prediction of the response to nCRT would be useful in LARC patient, but qualitative evaluation of response to treatment by medical images is not possible in early phase, because qualitative evaluation is performed by monitoring tumor anatomical characteristics, including length, area, and tumor volume, which are not apparent in early phases during therapy [9, 10]. Preprocessing is an important step in medical imaging, which is useful for improving quality, viewing of image details and increasing diagnostic accuracy by image enhancement, edge detection, noise reduction, etc. Filtering is usually applied in preprocessing, which is done by the Laplacian of Gaussian (LoG) filter. This filter highlights features at different scales, by two functions, including the Gaussian and the Laplacian function, for filtering and differentiation respectively. Low filter values is applied for highlighting fine anatomic details and high filter values is used for enhancing coarse anatomic details. Some studies have used different values of LoG filter in medical imaging of cancerous patients for better prediction of response process [9–11].

In a large number of high-throughput medical image features, radiomics are helpful to predict tumor behavior during therapy, providing accurate, noninvasive, and reliable biomarker for prediction of response to treatment [12, 13]. Feature selection algorithms are used for determination of relevant features to avoid over-fitting, resulted from more sample numbers compared with derived feature numbers, providing more accurate models. Quantitative texture analysis and data mining methods have been used as predictive and prognostic biomarkers in multiple cancers, including lung, breast, head and neck, and prostate cancer [14–18].

Machine learning (ML) is a programmable method, applied as predictive and prognostic models for radiomics which can “learn” using the data to improve response prediction. There are many different ML models for this purpose, which should be evaluated to determine the most useful model [19–21].

In this study, we aimed to investigate different ML algorithms on radiomic features extracted from rectal MRI to find best predictive models. Also, the effects of LoG filter and feature selection algorithms on LMs predictive performance were studied.

## Methods

**Fig. 1:**
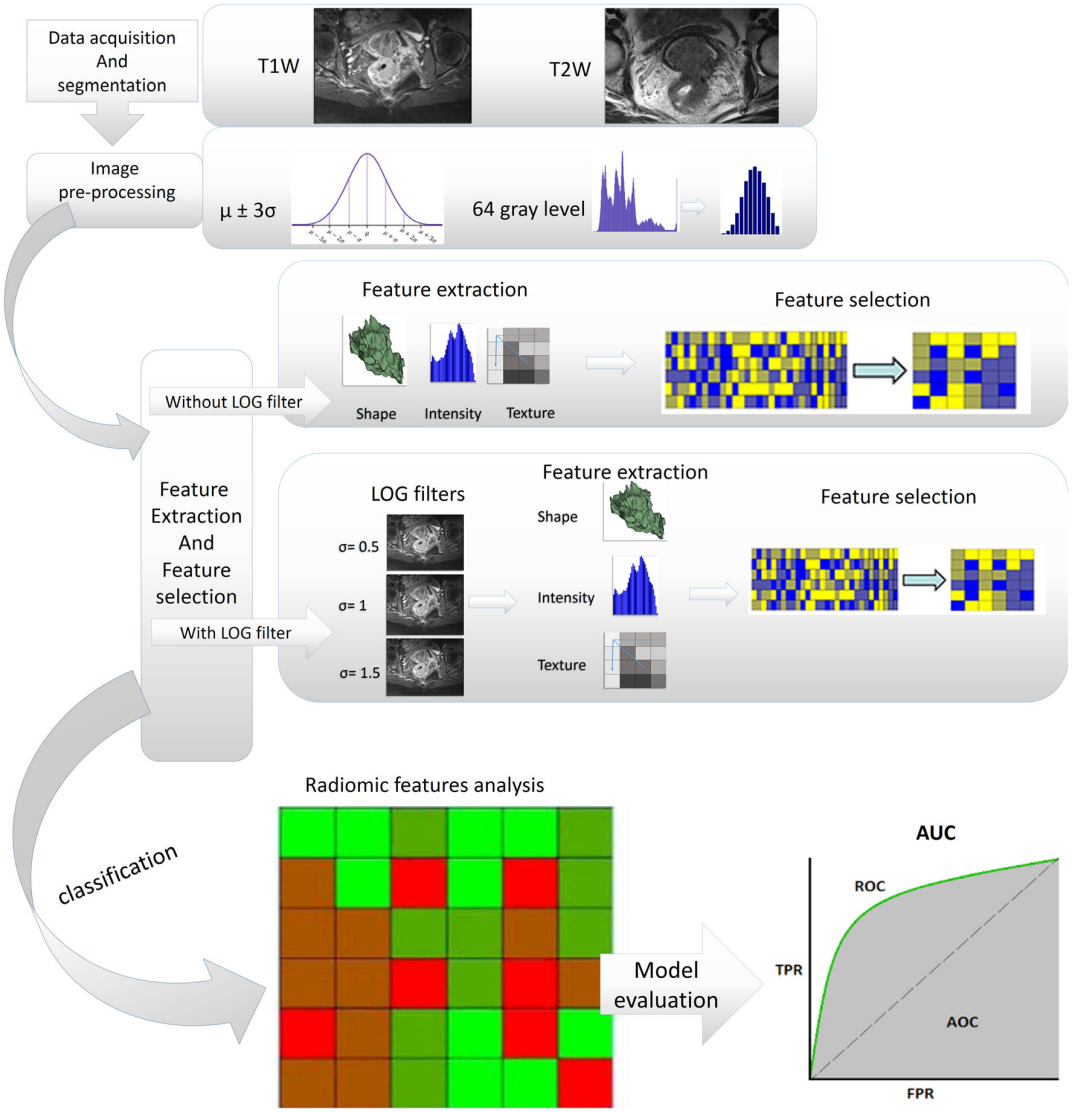
Overall framework of study

Figure 1 shows the different steps of our study in the format of overall framework. Below the different phases of study are outlined.

### Patient Characteristics

This study is retrospective, including 67 patients (44 male and 23 female) referred to Imam Khomeini hospital, Tehran University of medical science from October 2016 to April 2018.. This study was approved by the Ethics Committee of Iran University of Medical Science (ethics approval no. K17-137) and informed consent was acquired from all patients. Inclusion criteria were the location of tumor within 15 cm above anal verge, tumor penetration to perirectal fat (cT3–4) or lymph node involvement, age ≤ 80 years, WHO performance status of 0–2, normal CBC, liver and renal function tests, and lack of any prior treatment for the disease. All patients received concurrent nCRT. They received 45–46 Gy external beam radiation in 23–25 fractions with 18 MV photons to the tumor and loco regional disease including pre-sacral and internal iliac lymph nodes with a boost to the tumor for a total of 50–50.4 Gy, concurrent capecitabine at 825 mg/m2 twice daily. The exclusion criterion is as follows: patients with previous radiation therapy and/or chemotherapy for rectal cancer were excluded from study.

### Image Acquisition

All images were acquired on a 3.0-T MRI system (Tesla-Trio, Siemens Healthcare, Germany) 1 week before nCRT. A 32-channel pelvic phased array coil was used for signal reception. MRI protocol included axial, sagittal, and coronal T1-weighted images (TR 400 m/s, TE 17 m/s, FOV 20_20 mm, matrix 256_256, slice thickness of 3 mm with 1 mm gap) and axial, sagittal, and coronal T2-weighted images (TR 3000 m/s, TE 110 m/s, FOV 20_20 mm, matrix 256_256, slice thickness of 3 mm with 1 mm gap).

### Tumor Segmentation

Gross tumor volume was drawn around the rectal tumor, by two readers: a 10-year experience radiation oncologist, and a 15-year experience radiologist using a designated multi-platform, free, and open source software package for visualization and medical image computing (3D slicer, version 4.8.1; available at: http://slicer.org/). All slices were reviewed, followed by drawing on the T1W and T2W images. Tumor segmentation was performed on each MR image, creating a volume of interest (VOI).

#### Preprocessing and texture feature extraction

Pre-processing and discretization to 64 Gy level were done by a method proposed by Collewet et al. for noise reduction, intensity normalization, and discretization. In this method, all image intensities are normalized between *μ* ± 3*σ*, where *μ* is the mean value of gray levels inside the region of interest (ROI), and *σ* is the standard deviation [22, 23].

Also, feature extraction was applied on T1W and T2W MR Images with and without preprocessing filters in order to evaluate the filter effect on radiomic model performance. The filters include the LoG filter with sigma 0.5, 1, and 1.5. For feature extraction, we used the freely available radiomic software, imaging biomarker explorer (IBEX) that runs in Matlab platform.

Various radiomic features from different feature sets including intensity, shape-, and texture-based features were extracted from processed and un-processed T1W and T2W MR images. Extracted features included shape features (*n* = 17), intensity histogram features (*n* = 9), intensity direct (*n* = 19), neighbor intensity difference (*n* = 5), co-occurrence matrix features (COM) (*n* = 19), and gray level run-length matrix features (GLRLM) (*n* = 11) [9, 24].

#### Response Assessment

For all patients, surgery was done 4–8 weeks after nCRT. After inking, the specimens were fixed in formalin for 24 h. The whole tumor and mesorectum were serially sliced, axially, at 3 mm intervals, and treatment response was assessed according to the 4-category American Joint Committee on Cancer and College of American Pathologists (AJCC/CAP). The TRG according to AJCC/CAP was established as follows: grade 0 (pathologic response complete, PCR) is defined as no viable cancer cell; grade 1 (moderate response) means single cells or small groups of cancer cells; grade 2 (minimal response) are residual cancer outgrown by fibrosis; and grade 3 (poor response) is fibrosis outgrown by residual cancer [25–27].

#### Univariate Radiomic Analysis

For univariate analysis, significant radiomic features correlated with response were selected and a logistic regression classifier was used to find their predictive performance (based on AUC). Also, these features were compared between responder and non-responder groups. A paired *t* test was performed to assess the significance of the differences between two groups. Statistical significance was assumed if *p* < 0.05 and all reported *p*-values are two-sided.

#### Multivariate Radiomic Analysis

Statistical analyses were performed using the WEKA software version 3.8 (University of Waikato, Hamilton, New Zealand) [28]. Patients were divided into either the responder (Grade 0 or Grade 1) or non-responder (Grade 2 or Grade 3) group according to according to AJCC/CAP pathologic grading. We identify responder from a non-responder group by using 9 classifiers, including Bayesian Network, naive Bayesian network, Ada boost M1, iterative classifier optimizer, logit boost, randomizable filtered classifier, random sub space, random forest, and K logistic model tree (LMT). Then, effect of preprocessing LoG filters with 0.5, 1, and 1.5 values on these classification algorithms’ performance is investigated. For all of machine learning models the CfsSubsetEval (CF SUB E) feature selection algorithm was used. We compare model performance with the validation AUC using the 10-fold cross validation (CV).

### Feature Selection

Feature selection is the process of finding the most meaningful features, variables, and predictors for use in model construction. When the number of derived features is more than the number of samples, there is a danger of over-fitting analyses, and must be reduced by feature reduction [18, 29]. In this study, we investigate the effect of different feature selection techniques on classification performance using the WEKA software version 3.8 (University of Waikato, Hamilton, New Zealand). In the classifiers performance investigation with and without LoG filters, all of classifiers have the best performance in T2W MR Images with *σ* = 0.5. So, we investigate the effect of six different feature selection algorithms on classification model’s performance in T2W MR Images with *σ* = 0.5. The feature selection algorithms and their definition were shown on Table 1.

**Table 1:** Feature selection algorithms

Feature selection method	Definition
Cfs Subset Eval (CF SUB E)	Evaluates the worth of a subset of attributes by considering the individual predictive ability of each feature along with the degree of redundancy between them.
Correlation Attribute Eval (CO AT EV)	Evaluates the worth of an attribute by measuring the correlation (Pearson’s) between it and the class.
Gain Ratio Attribute Eval (GA FA AT)	Evaluates the worth of an attribute by measuring the gain ratio with respect to the class.
One R Attribute Eval (One R AT)	Evaluates the worth of an attribute by using the One R classifier.
Relief F Attribute Eval (RE F AT)	Evaluates the worth of an attribute by repeatedly sampling an instance and considering the value of the given attribute for the nearest instance of the same and different class.
Symmetrical Uncert Attribute Eval (SYM AT)	Evaluates the worth of an attribute by measuring the symmetrical uncertainty with respect to the class.

## Result

### Patients and Response

Sixty-seven patients (44 men; mean age, 60.01 years; age range, 31–80 years; 23 women; mean age, 52.2 years; age range, 27–67 years) with LARC were included in the study. All patients underwent simultaneous nCRT, followed by surgery. Patients’ CRT responses included 11 patients with Grade 0, 19 with Grade 1, 26 with Grade 2, and 11 with Grade 3 according to AJCC/CAP pathologic grading. Patient data and their response grade were shown in Table 2.

**Table 2:** Patient characteristics

Demographics	Frequency *N*	Percent %
Gender		
Male	44	65.7
Female	23	34.3
Total	67	100
Age		
18–40	15	22.4
41–60	23	34.3
> 61	29	43.3
Total	67	100
Response		
Grade 0	11	9.4
Grade 1	19	26.4
Grade 2	26	47.2
Grade 3	11	17

### Texture Analysis

**Fig. 2:**
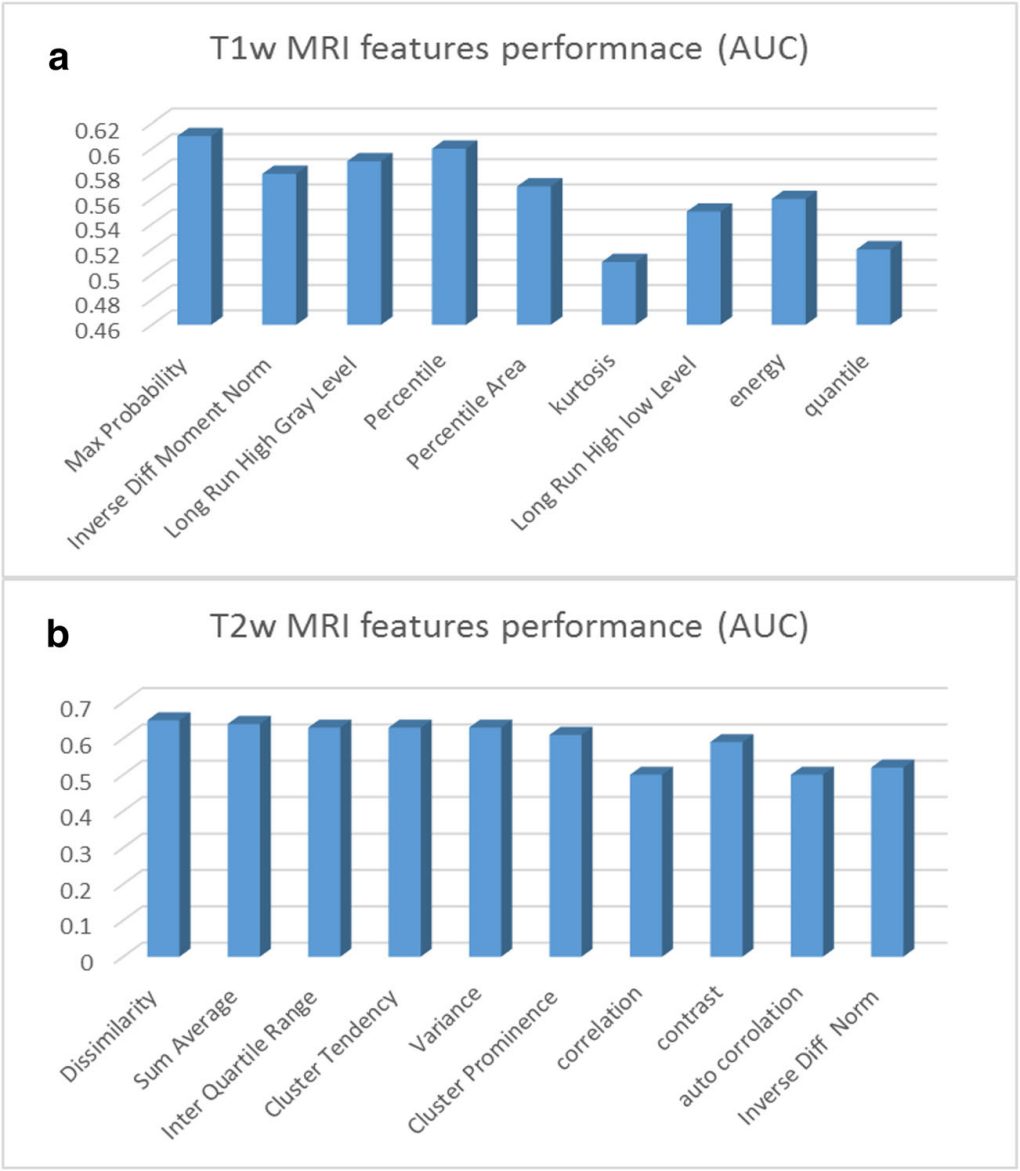
Features with high correlation ability to predict nCRT response in LARC in **a** T1W MR images. **b** T2W MR images

Radiomic features with high correlation to therapy response were selected for T1W and T2W MR images separately. Our univariate analysis showed that nine and 11 radiomic features have high correlation with nCRT response for T1W and T2W MR Image’s respectively. For T1W MR images, we found that three of the top radiomic features are from gray level co-occurrence matrix (GLCOM), two of them from gray level run length matrix (GLRLM) and four of them from intensity base feature set. For T2W MR Images, we found that all 11 top radiomic features are from co-occurrence matrix (COM) feature set. The results on AUC logistic regression classifier for these feature are shown in Fig. 2.

In T1W MR images, max probability has the best performance (AUC, 0.61/CI 0.55–0.66, *p* value 0.0004) followed by percentile (AUC, 0.60/CI 0.54–0.66, *p* value 0.0013), long-run high gray-level (AUC, 0.59/CI 0.53–0.61, *p* value 0.0031), inverse diff moment norm (AUC, 0.58), and percentile area (AUC, 0.57). In T2W MR images, dissimilarity has the best performance (AUC, 0.65/CI 0.58–0.71, *p* value, 0.0001), followed by Sum Average (AUC, 0.64/CI 0.58–0.70, *p* value, 0.0003), inter-quartile range (AUC, 0.63/CI 0.55–0.68, *p* value 0.0023), cluster tendency (AUC, 0.63), variance (AUC, 0.63), and cluster prominence (AUC, 0.61).

Based on these results in responder and non-responder groups, significant difference exists among selected radiomic features between two groups in TW1 and T2W MR images, but there is no significant difference between two groups in all features (*p* value > 0.05).

For multivariate radiomic analysis, our results were shown in Table 3. For ML classifiers performance investigation, BN and iterative classifier optimizer classifiers with AUC 0.64 (CI 0.57–0.68, *p* value 0.0003) and 0.72 (CI 0.61–0.77, *p* value 0.0001) was found as high predictive model for un-processed T1W and T2W MR Features respectively.

**Table 3:** AUC of texture feature analysis in different LoG filter values

		Classifiers								
		Bayesian network	Naive bayesian network	Ada boost M1	Iterative classifier optimizer	Logit boost	Randomizable filtered classifier	Random sub space	Random forest	K logistic model tree
	Filter value									
T1W	No filtration	63.9	51.2	50.0	51.0	51.2	50.0	52.2	51.3	50.0
	0.5 (fine)	52.3	54.2	56.8	58.1	53.8	53.2	59.1	55.3	50.0
	1 (medium)	56.2	68.0	78.1	70.3	78.1	52.5	79.3	53.1	52.9
	1.5 (coarse)	52.1	50.0	51.2	51.2	52.1	68	51.3	50.0	50.0
T2W	Filter value									
	No filtration	66.7	51.1	74.8	71.8	63.4	51.5	60.5	54.3	51.1
	0.5 (fine)	72.5	85.1	79.4	81.3	79.3	66.2	72.3	71.8	61.5
	1 (medium)	57.6	65.0	58.9	58.6	60.2	68.8	55.2	56.2	50.0
	1.5 (coarse)	57.6	80.6	80.1	53.6	80.1	67.1	66.4	57	60.9

Our result on pre-processed images showed that the LoG filter improves the classifiers performance. Results on pre-processed images with *σ* = 0.5, we also showed that random sub space (AUC, 59.1; CI 0.52–0.63, *p* value 0.0013) naive Bayesian network (AUC, 85.1, CI 0.77–0.89, *p* value 0.0001) classifiers have more predictive roles for T1W and T2W MR images respectively. In preprocessing with *σ* = 1, random sub space (AUC, 79.3, CI 0.75–0.83, *p* value 0.0023) and naive randomizable filtered classifier (AUC, 68.8) are the best predictive models for T1W and T2W MR images respectively. Also, results on pre-processed images with *σ* = 1.5, showed that randomizable filtered classifier (AUC, 68 CI 0.62–0.74, *p* value 0.0002) and naive Bayesian network (AUC, 80.6, CI 0.77–0.84, *p* value 0.0001) classifiers are the best predictive models for T1W and T2W MR Images respectively.

### Feature Selection Performance

In the last phase, the best result of LoG filters in T1w and T2w MR images is selected and the effect of different feature selection algorithms on mentioned classifiers performance is investigated. Almost the best performance was for T2w MR images with a fine (0.5) LoG filter. For effect of different feature selection algorithm analyses, our results were shown in Table 4. The best performance was for CF SUB E algorithm with a naive Bayesian network classifier (AUC, 85.1) followed by Ada boost M1 (AUC, 79.4), Logit Boost (AUC, 79.3), and K logistic model tree (AUC, 77.5). After CF SUB E feature selection algorithm the best performance was for SYM AT algorithm with logit boost classifier (AUC, 74.5).

**Table 4:** AUC of different feature selection algorithms in fine (sigma 0.5) LoG filter

	Classifiers								
Feature selection	Bayesian network	Naive Bayesian network	Adaboost M1	Iterative classifier optimizer	Logit Boost	Randomizable filtered classifier	Random sub space	Random forest	K logistic model tree
Cfs Subset Eval	72.5	85.1	79.4	81.3	79.3	66.2	72.3	71.8	77.5
Correlation Attribute Eval	61.2	57.8	73.1	55.9	75.6	72.0	55.7	51.6	58.8
Gain Ratio Attribute Eval	56.2	52.7	73.1	58.4	74.8	62.6	55.7	52.0	59.7
One R Attribute Eval	56.2	55.1	73.9	59.0	74.8	64.1	50.2	55.1	58.8
Relief F Attribute Eval	57.3	52.7	72.0	54.1	75.9	59.3	51.6	52.7	58.8
Symmetrical Uncert Attribute Eval	65.3	54.8	73.2	55.3	74.5	66.4	56.5	55.4	66.8

## Discussion

In oncology, imaging has a fundamental role, providing valuable data for cancer management. MRI is a noninvasive imaging modality, producing three-dimensional images, without ionizing radiation and better contrast and spatial resolution [30].

Radiomics, extracting and mining large number of quantitative and distinct medical imaging features, is a new field in medical imaging, which hypothesizes that some quantitative and distinct imaging features provide crucial information regarding tumor phenotype with clinical significance in different diseases, providing valuable data for personalized therapy [13, 31].

Our study demonstrated that quantitative features from Computerized Texture Analysis of LARC at Pretreatment T1w and T2w MR Imaging have correlation with response to nCRT and can be used as noninvasive biomarker for prediction of response to treatment. ML methods are reliable and accurate predictors and preprocessing LoG filter can improve their performance. In the majority of classifiers, performance of features derived from T2w was better than T1w MR images [32, 33].

Previous studies have shown the feasibility of radiomic modeling in LARC. Nie et al. using artificial neural network as classifier found that radiomic features extracted from T1/T2W, diffusion-weighted (DW) and dynamic contrast-enhanced (DCE) MR images could enhance the predictive power of pathologic response after preoperative nCRT for LARC [34]. In another study, Meng et al. used MRI texture analysis for nCRT response prediction and found several textures such as standard deviation (SD), kurtosis, and energy; and uniformity were statistically different between responder and non-responder groups [35]. Horvat N et al. used MR images to compare value of T2W radiomic textures compared with qualitative assessment at T2W and DW imaging for diagnosis of clinical complete response in patients with LARC after nCRT. They used random forest for classification and they found better performance of T2W-based radiomic features compared with qualitative assessment at T2-W and DW imaging for diagnosing pCR in patients with LARC after nCRT [36].

LoG filter is the combination of a Gaussian smoothing operator with a kernel of standard deviation (*σ*) followed by an isotropic Laplacian filter, which uses to highlight image details at various scales. In this study performance of ML methods improved by LoG filter in T2w and T1w MR images. Chee CG et al. used CT images without filtration and with LoG spatial filter with various filter values (1.0, 1.5, 2.0, and 2.5) to evaluate the association of texture features of LARC patients with nCRT response and disease-free survival (DFS). They found that responder group showed significantly lower entropy, higher uniformity, and lower standard deviation in no filtration and fine (1.0) and medium (1.5) LoG filter values than the non-responder group [9]. Also, Dinapoli N et al. used MR images with multiple σ of LoG filter to predict pCR probability using only pre-treatment MR images in LARC patients. They found that, only pre-treatment MR imaging can be helpful for predicting pCR probability in LARC patients [37].

The feature selection algorithm can identify relevant and robust features to improve model’s performance and avoid overfitting. At the last phase, we investigate the effect of six different feature selection algorithms (Table 1) on prediction process. Our results demonstrate that feature selection algorithm can affect the ML model’s performance and the best result was for CF SUB E algorithm. Megherbi et al. investigated the effect of clinical feature selection on surgery outcome predictions and as like as our study, they found effect of feature selection algorithm on ML performance [18, 38]. In another study, Saeys Y et al. investigated the necessity of applying feature selection techniques. They found that the main problem in the bioinformatics domain is the large input dimensionality, with the small sample sizes, and to deal with these problems, a wealth of feature selection techniques has been designed [18].

Although our results are significant, this study suffers from some limitations. First is the small sample size of 53 patients. Further studies with a large patient data are warranted to verify our results. Second is feature robustness and reproducibility. Based on several studies, radiomic feature are vulnerable against some challenges including image acquisition, reconstruction, segmentation, and processing. Although our data acquisition was same for all patients, there is a challenge on tumor segmentation. Third is classifier model validation. We used 10-fold cross validation which is proposed by several studies, but external validation with a large train data may improve the results.

## Conclusion

In conclusion, ML can be used as a response predictor model in LARC patients, but its performance should be improved. Effect of coarse preprocessing LoG filter on ML performance is better than fine filters and at the end, effect of feature selection algorithm on model’s performance is clear.
